# Drug-Coated Balloon Treatment of Very Late Stent Thrombosis Due to Complicated Neoatherosclerosis

**DOI:** 10.5935/abc.20160089

**Published:** 2016-06

**Authors:** Fernando Alfonso, Teresa Bastante, Javier Cuesta, Amparo Benedicto, Fernando Rivero

**Affiliations:** Hospital Universitario de La Princesa, Madrid - Spain

**Keywords:** Coronary Artery Disease, Percutaneous Coronary Intervention, Drug-Eluting Stents/adverse effects, Coronary Restenosis, Coronary Thrombosis/complications

## Abstract

We describe the treatment of a patient presenting with very-late stent thrombosis with the use of a drug-coated balloon. In this patient, optical coherence tomography disclosed that ruptured and complicated neoatherosclerosis was the underlying substrate responsible for the episode of very-late stent thrombosis. The potential use of drug-coated balloons in this unique scenario is discussed.

## Introduction

In-stent restenosis (ISR) and stent thrombosis remain major causes of stent failure^1-3^. ISR is usually a result of severe smooth muscle cell proliferation, but recent data suggest that neoatherosclerosis may also be the responsible pathological substrate^3^. Treatment of patients with ISR usually involves the use of drug-eluting stents or drug-coated balloons (DCB)^3^. Alternatively, stent thrombosis may occur as a result of sudden thrombotic occlusion of a previously patent stent or result from ruptured neoatherosclerosis with associated thrombosis. Treatment of stent thrombosis is very challenging and includes aggressive balloon angioplasty or repeat stent implantation^1^.

However, to the best of our knowledge, the use of DCB in patients presenting with stent thrombosis as a result of complicated neoatherosclerosis has not been previously reported.

## Case Report

**Figure 1:**
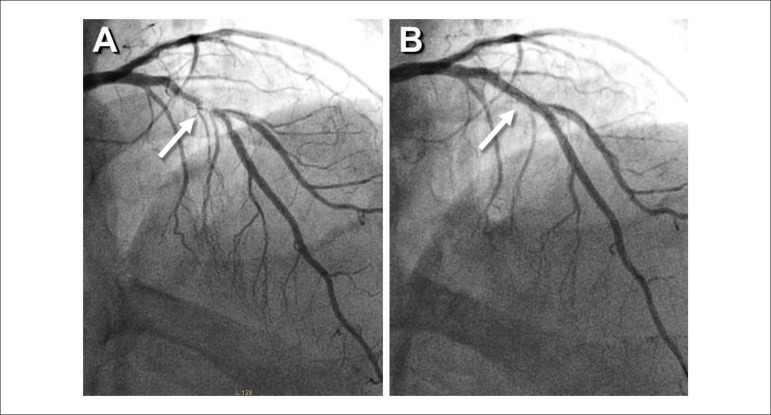
A) Coronary angiography with a cranial angulation showing a tight lesion (resulting in a luminal filling defect) in the mid part of the stent (arrow), on the proximal left anterior descending coronary artery that had a TIMI2 flow. B) Final result after DCB angioplasty.

**Figure 2:**
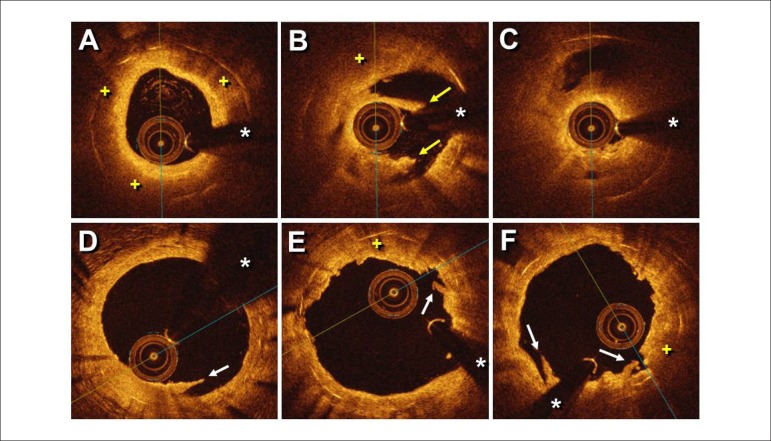
Optical coherence tomography images after thromboaspiration. A) Neoatherosclerosis with a glistening bright neointima and a heterogeneous pattern caused by large lipid pools (+). Notice that attenuation prevents adequate visualization of the underlying metallic stent struts. B) Ruptured neoatherosclerotic plaque (arrows). C) Occlusive neoatherosclerosis with thrombus. D, E, F) Optical coherence tomography images after DCB treatment. A large lumen was visualized along the entire stent length with some residual neointima, small dissections and residual thrombi (arrows). Confined residual lipid zones (+) were still recognized within the stent. (* = denotes wire artefact)

A 64-year-old man with hypercholesterolemia was admitted for a prolonged (3 hours) episode of chest pain at rest associated with nausea. Fourteen years before he had recieved a bare-metal stent for a severe lesion in the mid left anterior descending coronary artery. On admission, the ECG showed extensive T-wave inversion on the anterior leads. Urgent coronary angiography revealed a severe focal and eccentric lesion, with some haziness, at the mid segment of the stent, resulting in a TIMI 2 coronary flow (Figure 1A). Optical coherence tomography (OCT) disclosed a well-expanded and apposed stent, nicely covered by a thin ring of bright homogeneous neointima at the proximal and distal stent segments. However, neoatherosclerosis (glistening neointima overlying large lipid pools [ + ] shadowing the underlying stent struts) was readily recognized in the mid part of the stent (Figure 2A). In addition, a clear confined rupture of the fibrous cap was also identified (yellow arrows, Figure 2B) close to an occlusive lipid plaque associated with a large red thrombus (Figure 2C). Thromboaspiration was successful in improving the angiographic image and coronary flow, but only obtained a limited amount of red thrombus. High-pressure (22 bar) dilation with a noncompliant balloon yielded a good angiographic result. Then, a DCB (3 mm in diameter) was inflated for 60 seconds at this site, with an excellent final angiographic result and no images of residual dissections (Figure 1B). OCT confirmed a large lumen and thin residual neointima along the entire stent segment, but disclosed some minor intra-stent dissections (white arrows, Figure 2D, E, F) and some small residual laden thrombi at sites with residual neoatherosclerosis. The patient had an uneventful clinical outcome (peak troponin T 427 ug/L) and was discharged two days later.

## Discussion

Very-late stent thrombosis remains a rare, but devastating complication in patients undergoing percutaneous coronary interventions^1^. Recent studies suggest that neoatherosclerosis plays a major role in selected patients presenting with this feared complication^2^. Pathological studies suggest that neoatherosclerosis not only occurs more frequently but also earlier in patients treated with drug-eluting stents, as compared with those receiving conventional bare-metal stents^2^. Currently, OCT, with its unique resolution (15 *µ*m), represents the technique of choice for the diagnosis of neoatherosclerosis. Multiple studies have confirmed the importance of OCT in the diagnosis of neoatherosclerosis resulting in either in-stent restenosis or stent thrombosis^1,3^. Likewise, the use of DCB in patients presenting with in-stent restenosis has been well established^3^. Although neoatherosclerosis constitutes the underlying substrate in some of these patients, particularly in those treated with drug-eluting stents, the role of DCB in this specific anatomic subset remains to be elucidated. Our findings strongly suggest that DCB might also provide an attractive therapeutic strategy for selected patients with very late stent thrombosis as a result of neoatherosclerosis. Prospective studies are warranted to further define the potential role of this novel therapy in this challenging scenario.

## Conclusion

DCB constitutes an attractive therapeutic strategy for selected patients with stent thrombosis as a result of complicated neoatherosclerosis.
